# Comparative Evaluation of the Repair Bond Strength of Dental Resin Composite after Sodium Bicarbonate or Aluminum Oxide Air-Abrasion

**DOI:** 10.3390/ijms241411568

**Published:** 2023-07-17

**Authors:** Kinga Dorottya Németh, Roland Told, Péter Szabó, Péter Maróti, Réka Szénai, Zsolt Balázs Pintér, Bálint Viktor Lovász, József Szalma, Edina Lempel

**Affiliations:** 1 Department of Restorative Dentistry and Periodontology, University of Pécs Medical School, Tüzér Street 1, 7623 Pécs, Hungary; kinga.nemeth3@gmail.com (K.D.N.); simsreka@gmail.com (R.S.); 2 3D Printing and Visualization Centre, University of Pécs, Boszorkány Street 2, 7624 Pécs, Hungary; told.roland@pte.hu (R.T.); marotipeter8@gmail.com (P.M.); 3 János Szentágothai Research Center, Ifjúság Street 12, 7624 Pécs, Hungary; sz.piiit01@gmail.com; 4 Department of Oto-Rhino-Laryngology, Maxillo-Facial and Oral Surgery, St. John’s Hospital and North Buda Unified Hospitals, Diós Árok Street 1–3, 1112 Budapest, Hungary; pinter.zsolt.balazs@gmail.com; 5 Oral and Maxillofacial Department, Manchester Royal Infirmary Hospital, Manchester University Foundation Trust, Oxford Rd., Manchester M13 9WL, UK; balint10@hotmail.co.uk; 6 Department of Oral and Maxillofacial Surgery, University of Pécs Medical School, Tüzér Street 1, 7623 Pécs, Hungary; szalma.jozsef@pte.hu

**Keywords:** resin composite, repair bond strength, aluminum oxide, sodium bicarbonate, dental adhesive

## Abstract

The dental prophylactic cleaning of a damaged resin-based composite (RBC) restoration with sodium bicarbonate can change the surface characteristics and influence the repair bond strength. The purpose of this study was to compare the effect of sodium bicarbonate (SB) and aluminum oxide (AO) surface treatments on the microtensile bond strength (µTBS) of repaired, aged RBC. Bar specimens were prepared from microhybrid RBC and aged in deionized water for 8 weeks. Different surface treatments (AO air-abrasion; SB air-polishing), as well as cleaning (phosphoric acid, PA; ethylene-diamine-tetraacetic-acid, EDTA) and adhesive applications (single bottle etch-and-rinse, ER; universal adhesive, UA), were used prior to the application of the repair RBC. Not aged and aged but not surface treated RBCs were used as positive and negative controls, respectively. The repaired blocks were cut into sticks using a precision grinding machine. The specimens were tested for tensile fracture and the µTBS values were calculated. Surface characteristics were assessed using scanning electron microscopy. AO-PA-UA (62.6 MPa) showed a 20% increase in µTBS compared to the NC (50.2 MPa), which proved to be the most significant. This was followed by SB-EDTA-UA (58.9 MPa) with an increase of 15%. In addition to AO-PA-UA, SB-EDTA-UA could also be a viable alternative in the RBC repair protocol.

## 1. Introduction

Resin-based composites (RBC) are the most frequently used dental restorative materials. Although the survival rate of direct RBC restorations is high, ranging between 88 and 98%, the failures that may occur can be related to the high level of chemical, mechanical, and physical stress in the oral cavity [1,2,3]. Most clinical deteriorations are due to defects in restorations and lesions of secondary caries along the restoration margins. The occurrence of a limited defect of the restoration does not necessarily mean an irreversible failure and does not require the immediate, complete replacement of the restoration. The clinician has an opportunity to implement some form of minimal intervention treatment to correct the defect. Minimal intervention includes the correction of a superficial deterioration and the repair of an accessible, localized defect [4]. The main advantage of such approaches is the preservation of sound tooth tissues and functionality, which can extend the life expectancy of the restoration and slow the “restorative death spiral” [5,6]. There are a few factors which play a role in the decision to repair rather than replace a restoration, such as patient’s caries risk status, the clinical condition of the restored tooth, the medical history of the patient and cost/benefit assessments, among others [6]. Consequently, the success and survival of the repaired restoration depends on a prudent management plan and decision-making process based on risk assessment. This includes the assessment of further caries, structural deterioration, catastrophic failure, and loss of pulp vitality. The repair of RBC with the addition of restorative material is a minimally invasive approach and is indicated in cases of localized shortcomings that are clinically unacceptable [5]. During repair, the defective part of the restoration or adjacent tooth tissue is removed and replaced by the repair RBC [6]. Since the repair of an existing RBC restoration is indicated after months or years of service life, the elution of reactive components, water absorption and chemical, physical and enzymatic degradation may make repair complicated [7,8]. These issues may compromise the durable bond between the aged and repaired RBC. While the optimal adhesion between RBC layers is chemical, through the bonding of new monomers to the matrix and/or exposed fillers of the old RBC, the bonding of new to old RBC is, for the most part, micromechanical via the penetration of the new monomers into the irregularities of the repaired RBC [9,10,11]. Hence, an increase in the substrate RBC’s surface area is desirable in an attempt to optimize the repair bond strength. Surface treatment also allows for direct chemical interactions with unconverted C=C bonds and the deeper penetration of the adhesive into the irregularities, which are both advantageous to increase adhesion [12]. Although numerous surface treatment methods have been reported, none of them have been accepted as the gold standard. Mechanical surface treatment is achieved by grinding with diamond burs or by air-abrasion, creating a micro-retentive surface that enables the interlocking of the new RBC [9,13]. In addition, the removal of the chemically altered and contaminated superficial layer may enhance the surface energy of the reparable RBC [12]. While air-abrasion with aluminum oxide (Al_2_O_3_) particles or silica coating is the most widely investigated and recommended surface treatment method [12,14], there are general dental practitioners who may not have proprietary silica coating devices or aluminum oxide particulate abrasives. Many practices apply prophylactic airborne powders for stain removal and the cleaning of tooth surfaces, either with calcium carbonate or sodium bicarbonate for air-polishing. Before the repair of a defective RBC restoration, it could be an option to clean the surface with one of these abrasive powders. Several studies have proved that the use of these prophylactic airborne particles can increase the surface roughness (RA) of RBC restorations by leaving large depressions on the surface [15,16,17]. Therefore, it is suggested that air polishing should be avoided on all types of RBCs [17]. The mean value of RA as a result of a prophylactic air-polishing can range between 0.3 and 10 µm, and depends on the type of applied powder, exposure time, pressure, and the type of the recipient RBC [17,18,19,20]. However, according to our best knowledge, there is no report in the literature about the effect of sodium bicarbonate air-polishing on the microtensile bond strength (μTBS). Therefore, in this in vitro study we attempt to simulate a real clinical scenario, where the defected RBC restoration is cleaned with sodium bicarbonate air-polishing before repair with a new RBC, and measure its effect on the μTBS. The aim was to compare the effect on μTBS of sodium bicarbonate air-polishing with the generally recommended aluminum oxide air-abrasive surface treatment. An additional objective was to investigate the cleaning effect of phosphoric acid as a strong acid or EDTA as a chelator on μTBS after surface treatment with the above-mentioned particles. The null hypotheses of our investigation were the following: (a) there is no difference in μTBS using sodium bicarbonate or aluminum oxide airborne particles before RBC repair; (b) there is no effect of using phosphoric acid or EDTA as a cleaning method on the repair μTBS; (c) there is no additional effect of the application of adhesive system on the repair μTBS.

## 2. Results

### 2.1. Microtensile Bond Strength Testing

**Figure 1:**
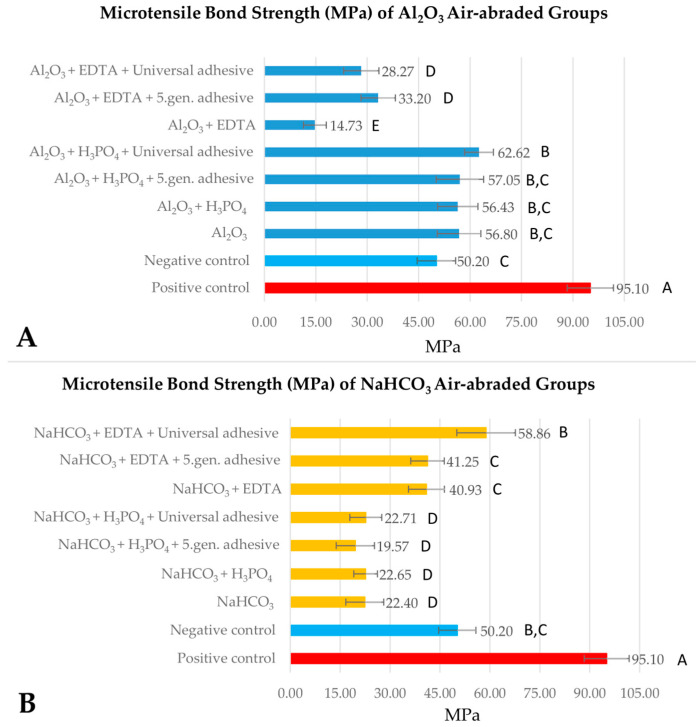
Comparison of microtensile bond strength among the aluminum oxide air-abraded (**A**) and the sodium bicarbonate air-polished (**B**) groups. Different capital letters denote statistically significant differences among the compared aluminum oxide air-abraded and sodium bicarbonate air-polished groups, analyzed by one-way analysis of variance (ANOVA) and Tukey’s post hoc test.

The immediately repaired positive control group (group 1) exhibited significantly higher µTBS (95.1 ± 6.7 MPa) than the aged groups pretreated with the different protocols (*p* < 0.001). The aged but not surface-conditioned negative control (group 2) showed 52.8% of the value (50.2 ± 5.6 MPa) observed with the positive control. The results of the microtensile bond strength tests of the aluminum oxide air-abraded groups (group 3–9) compared to the positive and negative groups are presented in Figure 1A. Aluminum oxide air-abrasion (group 3) and air-abrasion followed by phosphoric acid cleaning (group 4–6) provided similar results to the negative control. The highest µTBS (62.6 ± 4.2 MPa) of 65.8% of the positive control was achieved with the use of phosphoric acid cleaning and universal adhesive application (group 6). The repair bond strength showed a 20% increase compared to the negative control. Aluminum oxide air-abrasion followed by EDTA cleaning, with or without adhesive (groups 7–9), showed the lowest µTBS, with values of 15.5–34.9% of the positive control group.

**Figure 2:**
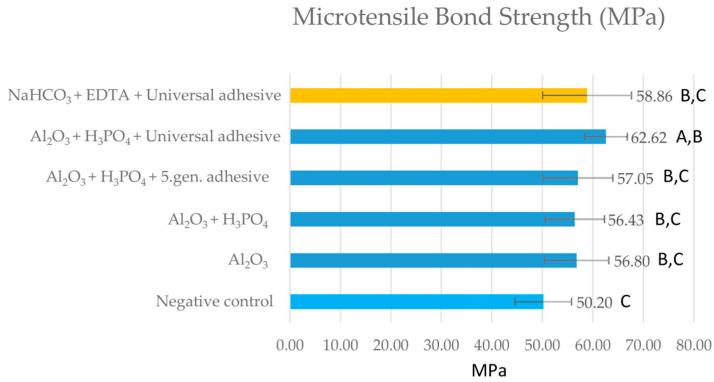
Comparison of microtensile bond strength among those aluminum oxide and sodium bicarbonate air-abraded groups, which achieved higher values than the negative control. Different capital letters denote statistically significant differences among groups analyzed with one-way analysis of variance (ANOVA) and Tukey’s post hoc test.

The results of the µTBS tests of the sodium bicarbonate air-polished groups (group 10–16) compared to the positive and negative groups are presented in Figure 1B. Compared to the negative control, significantly lower µTBS values were detected in several air-polished groups (group 10–13), except for the EDTA-cleaned groups (group 14–16), where the measured µTBS values did not differ statistically significantly (*p* > 0.05). µTBS values of group 10–13 were only 15.5–26.2% of the positive control group, and meanwhile group 14–15 provided ~45.5% of the positive control group. The application of universal adhesive after sodium bicarbonate air-polishing and EDTA cleaning (group 16) led to a bond strength value of 65% of the positive control. Although this represented a ~15% increase compared to the µTBS value of the negative control, it was not statistically significant. Those aluminum oxide (group 3–6) and sodium bicarbonate (group 16) surface conditioned groups that achieved higher values in comparison to the negative control were subjected to another statistical analysis (Figure 2). The results showed that cleaning with EDTA and using a universal adhesive on sodium bicarbonate air-polished samples resulted in similar µTBS to alumina air-polished samples, except for those cleaned with EDTA.

Table 1 shows the relative effect size of the independent factors, such as surface treatment (ST), adhesive system (AS) and cleaning method (CS), and their interactions on the microtensile repair bond strength of the investigated RBC groups. Factors of ST, ST × CM and AS showed a statistically significant effect on µTBS. While the effect size of the ST factor and its interaction with cleaning method (ST × CM) was found to be moderately large, the effect size of the other investigated factors and their interaction was negligible. The observed power showed the probability of correctly rejecting the null hypothesis. The power of the ST and ST × CM effect was 1.000. If the study was to be replicated 100 times, the null hypothesis would be correctly rejected on 100% of those replications. For AS, the null hypothesis would be correctly rejected on 64.1% of the replications, and for its interactions with CM and ST, this value is 37.7% and 28.3%, respectively. The observed power for the interaction of the three evaluated effects was 30.1%.

### 2.2. Failure Type Analysis

**Figure 3:**
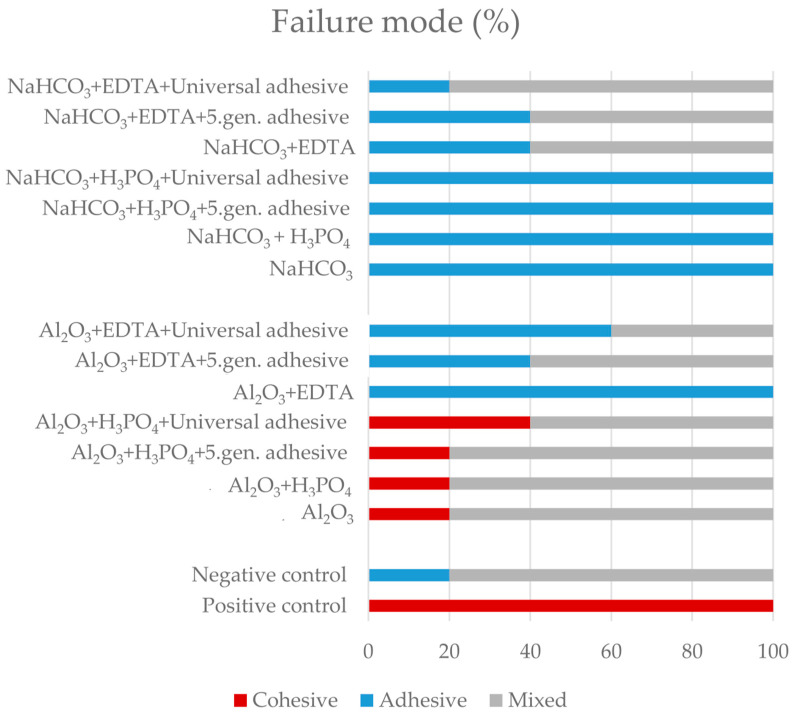
Failure modes of the investigated groups. Cohesive failure type represents failure within the substrate or the repair RBC, adhesive failure occurs between substrate and repair RBC, and both cohesive and adhesive failures are observable in mixed failure mode.

The failure mode distribution is shown in Figure 3. Adhesive and mixed failures were predominately detected. A cohesive failure mode of 100% was noted in the positive control group. The cohesive failure mode was also observed in 20–40% of samples pre-treated with aluminum oxide cleaned with phosphoric acid (group 3–6). In groups which were treated with sodium bicarbonate, the most frequent failure mode was adhesive (group 10–13), except the EDTA treated groups (group 14–16), where mixed failure was predominately detected.

### 2.3. Scanning Electron Microscopic Analysis

**Figure 4:**
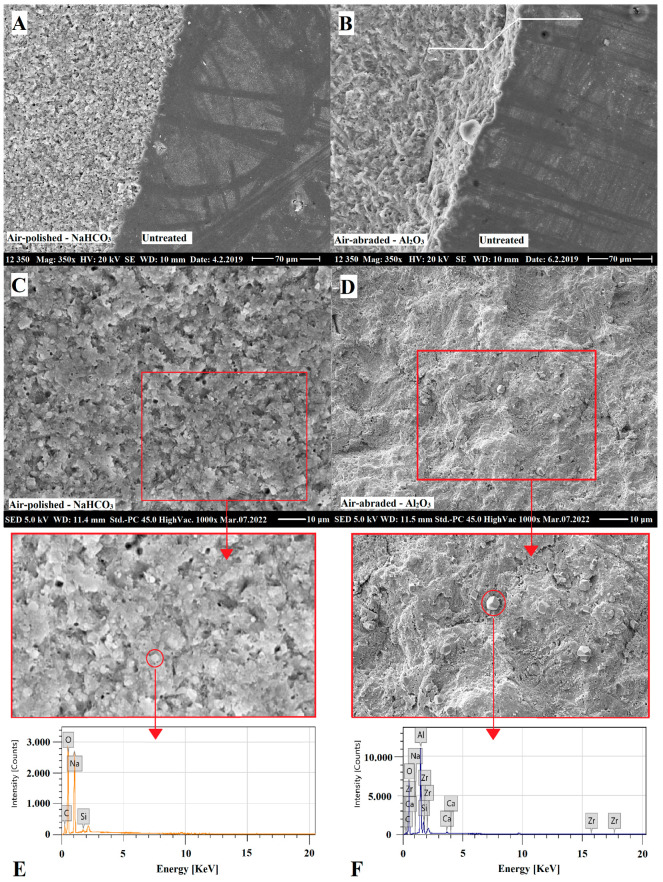
Representative scanning electron microscopy images of air-polished (65 µm sodium bicarbonate, NaHCO_3_) and air-abraded (53 µm aluminum oxide, Al_2_O_3_) resin-based composite (RBC) samples (Filtek Z250, 3M ESPE, St. Paul, MN, USA). The magnification of images (**A**,**B**) is 350×, where half of the RBC specimen surface was covered with a metal strip to avoid surface treatment and the uncovered was air-polished/air-abraded. Note the depth discrepancy (indicated with double broken white line) between Al_2_O_3_ treated and untreated parts of the sample (**B**), which proves the significant removal of the surface layer. Images (**C**,**D**) show the 1000× magnification of NaHCO_3_ air-polished and Al_2_O_3_ air-abraded RBCs. Images (**E**,**F**) represent the energy-dispersive X-ray elemental analysis of remnant particles (highlighted by red circles on enlarged image details in red boxes) of NaHCO_3_ and Al_2_O_3_, respectively.

**Figure 5:**
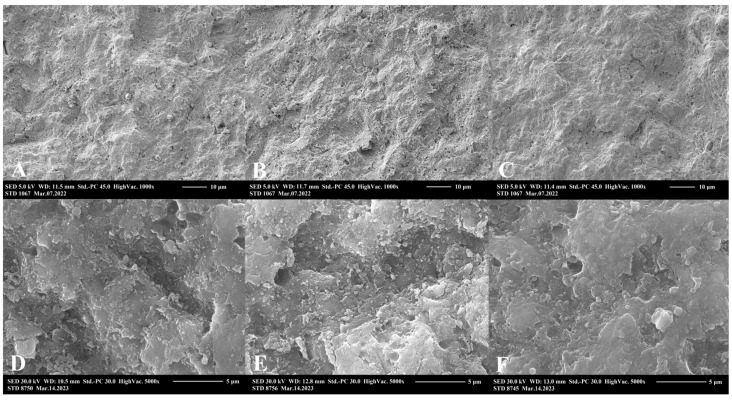
Representative scanning electron microscopy images at 1000× (**A**) and 5000× (**D**) magnification of aluminum oxide air-abraded resin composite (Filtek Z250, 3M ESPE, St. Paul, MN, USA) surface; images at 1000× (**B**) and 5000× (**E**) magnification of aluminum oxide air abraded surface cleaned with 35% phosphoric acid; images at 1000× (**C**) and 5000× (**F**) magnification of aluminum oxide air-abraded surface cleaned with ethylene diamine tetra-acetic acid.

**Figure 6:**
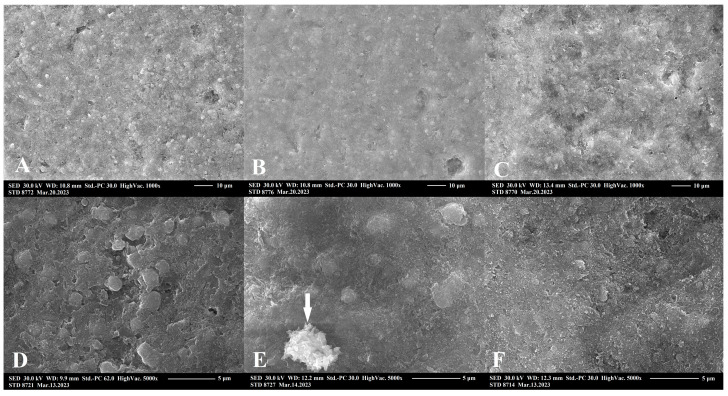
Representative scanning electron microscopy images at 1000× (**A**) and 5000× (**D**) magnification of sodium bicarbonate air-abraded resin composite surface; images at 1000× (**B**) and 5000× (**E**) magnification of sodium bicarbonate treated surface cleaned with 35% phosphoric acid, white arrow shows a crystalline pollutant; images at 1000× (**C**) and 5000× (**F**) magnification of sodium-bicarbonate-treated surface cleaned with ethylene diamine tetra-acetic acid.

SEM surface morphology analysis showed a significant roughening by aluminum oxide air-abrasion, and a less significant but unequivocal surface area increase in the case of sodium bicarbonate conditioning (Figure 4). A descriptive analysis revealed the destructive and non-selective effects of aluminum oxide air-abrasion on both resin matrix and filler particle compartments of RBC. Meanwhile, sodium bicarbonate air-abrasion was destructive, mostly on the resin matrix, leaving intact, rounded filler particles embedded in the matrix network. Both surface treatment methods resulted in remnant abrasive debris deposits on the RBC surfaces. EDS elemental analysis measured the composition and concentration of metallic and non-metallic elements of the region of interest. On the surface of the untreated RBC, the following elements were detected (given in atom% ± S.D.): O (48.2 ± 0.3), C (30.8 ± 0.3), Si (17.5 ± 0.05), Zr (3.0 ± 0.02), Na (0.2 ± 0.02) and Al (0.2 ± 0.01). There was no significant difference in general composition between untreated and treated samples. However, on the sodium bicarbonate air-polished samples particles with high concentration of Na, the following compositions were demonstrated (given in atom% ± S.D.; n = 5/group): O (56.6 ± 0.3), Na (23.2 ± 0.2), C (20.1 ± 0.2) and Si (0.2 ± 0.02) (Figure 4E). On the surfaces treated with aluminum oxide, aluminum-rich residual grains with the following composition were found (given in atom% ± S.D.): O (68.3 ± 0.3), Al (15.5 ± 0.1), C (12.9 ± 0.2), Si (2.8 ± 0.03), Na (0.2 ± 0.02), Zr (0.2 ± 0.01) and Ca (0.2 ± 0.01) (Figure 4F). Figure 5 demonstrates representative images of aluminum oxide air abraded surfaces at 1000× and 5000× magnification. Cleaning with either 35% phosphoric acid (Figure 5B,E) or EDTA (Figure 5C,F) after aluminum oxide air-abrasion seemed to remove the remnant grains of air-abrasive powder. However, the surface after phosphoric acid treatment additionally looked rougher at 1000× magnification, and this perception was not experienced at a higher (5000×) magnification. Cleaning with EDTA resulted in a smoother surface topography, which was clearly visible both at 1000× and 5000× magnification. Figure 6 demonstrates representative images of sodium bicarbonate air-polished surfaces at 1000× and 5000× magnification. Cleaning with EDTA (Figure 6C,F) resulted in a rougher appearance of sodium bicarbonate treated samples since seemingly nano-erosive depressions increased the surface.

## 3. Discussion

Sodium bicarbonate air-polishing is a widely applied alternative to conventional oral hygiene maintenance therapy used to remove deposits from the tooth surface. However, it can also roughen the RBC surface, which can even be favorable during RBC repair [21,22]. Since no data are available in the literature regarding the effect of sodium bicarbonate surface treatment in RBC repair, this in vitro study analyzed the µTBS and surface topography of repaired RBC after air-polishing with sodium bicarbonate compared to the aluminum oxide air-abraded RBCs. In addition, this study tested the effect of phosphoric acid or EDTA cleaning, and additionally analyzed the influence of different adhesive systems on repair bond strength. Our results showed that the surface characteristics and µTBS were dependent on the surface conditioning method, and also varied in relation to each cleaning method and adhesive system used, yielding the rejection of all null hypotheses.

In this study, different RBC repair approaches were evaluated and a µTBS test was used to measure the repair bond strength. An advantage of this test is a better stress distribution during the loading of the bonded interface of small samples, thus reducing the frequency of cohesive fractures [23].

For a successful repair, the bonding interface between the existing resin and the repair RBC should provide a bond strength similar to the cohesive strength of the substrate RBC. However, none of the surface treatment protocols tested on aged RBCs were capable of generating similar bond results after repair compared to those achieved on the samples of the non-aged, non-conditioned positive control group. This was demonstrated in other investigations, as well [24,25]. According to a systematic review and meta-analysis, only 20.8% of the applied surface treatments could approach a bond strength similar to the cohesive strength of the original RBC [26]. The cohesive strength of the Filtek Z250 positive control was measured to be 95.1 MPa. This value was similar to measurements from other studies where the substrate cohesive strength was 104.3 MPa [27]. Bonding between two freshly connected RBC layers was achieved in the presence of an oxygen-inhibited layer of unpolymerized resin, which readily adapted the overlaying RBC and formed an interdiffused zone where copolymerization can take place to produce chemical bond [28]. However, when the RBC needs repair, the surface no longer has an oxygen inhibition zone, which leads to decreased interfacial bond strengths [29]. During our experiment, aged RBC specimens were used to test the µTBS after different surface conditioning methods. By aging the samples, it was possible to partially simulate changes that may occur in the RBC during clinical service, such as a reduction in unreacted methacrylate groups, water sorption and the leaching of components, among others [7]. An important aspect of this study is the inclusion of a negative control, as it is essential to compare and relate the effect of various repair techniques. However, many prior investigations did not include a negative control for comparison [8,30,31]. The repair bond strength of the aged but not surface conditioned samples (negative control) reached only half of that of the positive control. Contrary to expectations, almost all surface conditioned groups showed statistically similar or lower µTBS compared to the negative control (50.2 MPa). Only Group 6 (aluminum oxide air abrasion, phosphoric acid cleaning, universal adhesive application) showed statistically higher µTBS. The results of other studies are not consistent regarding the bond strength of the investigated surface conditioning protocols compared to the negative control. Similar to our findings, in another study, no statistically significant difference was detected between most of the investigated groups and the aged but not surface treated samples [32]. In contrast, other investigations demonstrated that most surface treated samples significantly exceeded the µTBS values of the negative reference, regardless of the conditioning protocol [11].

On comparison of the effect of bonding to aged RBC, our results showed that air-polishing with sodium bicarbonate without chemical cleaning and adhesive application could not achieve the bond strength provided by the aluminum oxide air-abrasion. This is contrary to findings from orthodontic investigations, where the bond strength of ceramic brackets to RBC restorations after sodium bicarbonate air-blasting was found to be superior compared to aluminum oxide air-abrasion or other mechanical and chemical methods [33,34,35]. Supporting the observed µTBS values in this study, SEM images showed an irregular topography pattern after surface treatment using 53 μm aluminum oxide. Structural changes, such as the destruction of both the resin matrix and filler particles, may lead to a better micromechanical interlocking between the substrate and repair RBC after surface treatment with aluminum oxide particles [36]. Aluminum oxide as the standard abrasive powder for use in air-abrasion has a Mohs hardness index of 9, which is strong enough to remove the contaminated superficial layer of RBC via the non-selective destruction of the RBC compartments (see Figure 4 and Figure 5). Additionally, their irregular shape and rough edges may be abrasive [37]. Several investigations proved the beneficial effect of aluminum oxide air-abrasion in repair bond strength with a range of µTBS values from 40 MPa to 90 MPa [12,36,38,39]. Although our µTBS values achieved by aluminum oxide air-abrasion fell within this range, the repair bond strength could not significantly exceed the value of the non-treated negative control. According to the results of other research, the surface treatment was only secondary in terms of repair bond strength and aluminum oxide air-abrasion did not result in better bonding compared to other mechanical surface treatment methods [11].

On the other hand, air-polishing with 65 μm sodium bicarbonate resulted in surface roughening via the selective removal of the softer resin matrix only, leaving the filler particles intact. These are covered by resin, showing that sodium bicarbonate with a Mohs hardness of 2.8 [40] was not able to destroy or break the zirconia-silica fillers (see Figure 4 and Figure 5). This only caused some of the looser filler particles to turn out from the surface. Although micromechanical retention on the aged RBC substrate is a key mechanism to achieve reliable repair bond strength [11,41], there is still no consistent correlation between the roughness profile of the conditioned RBC surfaces and the bond strengths achieved [9,38]. According to the results found by Wendler et al., macro-retentive, highly rough and irregular surfaces do not necessarily mean higher repair bond strength, compared to a more homogenous but micro-retentive surface [11], which has higher total surface area for adhesion [12]. 

In this study, besides the significant effect (*p* < 0.001) observed for surface treatment on the repair bond strength with a 0.516 partial eta-squared value, the interaction of a cleaning method with mechanical surface treatment also had a significant impact (*p* < 0.001), with a 0.641 partial eta-squared value. The effect of two chemical agents, 35% phosphoric acid and 15% EDTA, on the bond strength was also compared after aluminum oxide air-abrasion and sodium bicarbonate air-polishing. Since the phosphoric acid conditioning of enamel and dentin adjacent to the reparable RBC is a routine step to achieve adequate bond strength on the tooth, the etching of the substrate RBC prior to repair is a widely applied method to remove debris and grinding dust [10,30,42,43]. Based on our results, phosphoric acid etching did not result in a significant increase in repair bond strength, either after aluminum oxide air-abrasion or after sodium bicarbonate air-polishing. There are contradictions in the literature regarding the benefit of cleaning with phosphoric acid during surface conditioning. Similar to our findings, phosphoric acid had no effect on the surface characteristics of hybrid and nanofilled RBCs [44] or provided clinically unacceptable bond strengths in hybrid or microfilled RBCs [45]. In contrast, other investigation found that, among the assessed conditioning methods, aluminum oxide air-abrasion followed by phosphoric acid etching produced the highest bond strength [46]. It is supposed that phosphoric acid may improve the surface characteristics and lead to an improved wettability, promoting adhesion to the aged material [11,32]. Cleaning with EDTA resulted in a contradictory effect on the aluminum oxide air-abraded samples compared to the sodium bicarbonate treated specimens. µTBS values were significantly reduced by EDTA on aluminum oxide air-abraded RBCs, and meanwhile the opposite was observable on the sodium bicarbonate treated samples. According to the SEM analysis, sodium bicarbonate debris particles were observed on the air-polished RBC substrate, which may have wedged into the surface. EDTA as a weak acid can react with sodium bicarbonate and forms carbonic acid, which in turn decomposes to water and carbon dioxide gas. While the EDTA complexes with sodium ions are weak compared to those formed with many other metal ions [47], this complexation may be important in RBC repair using sodium bicarbonate for surface treatment, since it is water-soluble. Hence, it becomes easier to wash off compared to sodium bicarbonate particles stuck to the surface. In addition, any surface roughness or irregularities of nanoscale size were clearly observed to be superimposed over the surface depressions on EDTA etched specimens compared to the non-etched samples. In contrast, the EDTA cleaning of the aluminum oxide air-abraded RBCs resulted in a clearer but less retentive appearance, as observed on SEM images, which was supported by the results of the µTBS tests.

The application of a bonding system yielded the best bond strength results in several investigations, even reaching the cohesive strength of the RBC [9,48,49,50]. The positive effect of adhesives lies in the fact, that due to their good penetration ability, they infiltrate the microstructure more easily, compared to a high-viscosity repair RBC [32,51]. In this study, a fifth-generation adhesive (Adper Single Bond 2) containing both the primer and the adhesive was compared to a universal adhesive containing 10-MDP and silane. The general linear model revealed a statistically significant effect of the adhesive system on the repair bond strength; however, the effect size was low. The highest repair bond strength was detected in samples which had been aluminum oxide air-abraded followed by phosphoric acid cleaning and the application of universal adhesive. The universal adhesive improved the repair bond strength of sodium bicarbonate air-polished and EDTA-cleaned samples, too, to the extent that it was statistically close to the best performing groups; however, its use did not result in statistically significantly different values compared to groups without the universal adhesive. The application of universal adhesive on sodium bicarbonate and EDTA-treated RBC surfaces improved the µTBS by 15% compared to the negative control group, which may be useful from a clinical point of view if the clinician cleans the defected RBC surface with sodium bicarbonate air polishing before repair. Most of the universal adhesives contain 10-MDP, which has been identified as being capable of establishing an intensive and stable chemical interaction with metals, zirconia and dental tissues by forming insoluble calcium salts [52,53,54]. The tendency for a higher bonding force with universal adhesive can be explained by the presence of zirconium silicate as fillers of the RBC (Filtek Z250) used in the present study. This result is in line with other research which investigated the repair bond strength of CAD/CAM resin-matrix ceramics containing zirconia silicate fillers [55]. Additionally, 10-MDP was demonstrated to improve the repair bond strength of low-viscosity bulk-filled RBC with barium-alumino-fluoro-borosilicate and strontium alumino-fluoro-silicate glass content [48,49]. In addition to 10-MDP, the Scotchbond Universal Plus Adhesive tested here had silane in its formulation, which theoretically would allow bonding to acid-sensitive inorganic components and facilitates the infiltration of RBC in microscopic grooves resulting from a prior surface preparation, which may also contribute to the tendency for better repair bond strength [45,50,56]. Based on the results of this study, the generalized linear model revealed an insignificant effect (*p* > 0.05) of adhesive application. In contrast, another investigation found that the application of an intermediate bonding system was a key element in achieving reliable repair bond strengths, whereas the kind of mechanical surface treatment appeared to play a secondary role [11].

One of the limitations of the present study is the use of a single substrate RBC to test the repair methods. Several research works concluded that the type of the substrate RBC used has a significant role in the repair bond strength [12,27,39], and thus further examinations are necessary, with inclusion of more RBC as both substrate and repair RBC. Another limitation may be the use of sodium bicarbonate with only one particle size, of 65 µm, in the present study. Increased roughness resulting from kinetic abrasion is influenced by the particle’s characteristics, such as particle size, hardness and angularity [57]. Depending on the particle size, the surface roughening effect of different brands of sodium bicarbonate has been reported to be dissimilar [58]. Further studies are necessary to measure the effect of different particle sizes and prophylactic air-polishing powders on the repair bond strength.

## 4. Materials and Method

### 4.1. Specimen Preparation

The used materials and equipment are listed in Table S1. The chemical composition and manufacturer’s details for the materials (RBC, adhesives, surface cleaners) used in the study are presented in Table 2.

As a first step of the sample preparation, RBC blocks were prepared (n = 16) to simulate the old restoration to be repaired. Thereafter, the blocks were subjected to the aging procedure, followed by surface conditioning prior to repair with the “new” RBC. 

**Figure 7:**
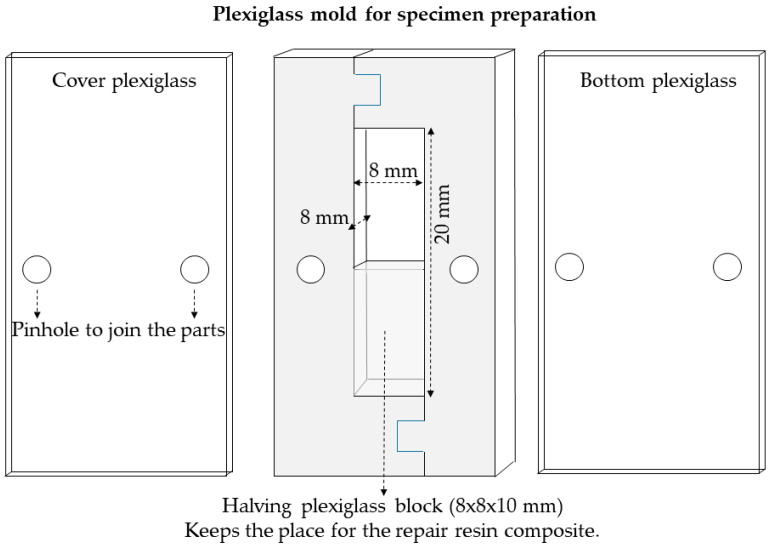
Custom-made laser cut transparent thermoplastic poly(methyl-methacrylate) (plexiglass) template used for sample preparation.

A custom-made laser cut transparent thermoplastic poly(methyl-methacrylate) (Perspex, Chelmsford, UK) template with an inner dimension of 8 mm height, 8 mm width and 20 mm length was used for sample preparation (Figure 7).

A2 shade RBC specimens were prepared by condensing the material in 1.5 mm thick increments into half (10 mm in length) of the template to represent the restoration to be repaired. All increments were irradiated with the same Light Emitting Diode (LED) curing unit (LED.D, Woodpecker, Guilin, China; average light output given by the manufacturer 850–1000 mW/cm^2^; ʎ = 420–480 nm; 8 mm exit diameter fiberglass light guide) in standard mode for 40 s from each surface, powered by a line cord. The radiant exitance (mW/cm^2^) was measured using a checkMARC radiometer (Bluelight Analytics, Halifax, NS, Canada). The room for the repair RBC was provided by an 8 × 8 × 10 mm^3^ plexiglass block in the other half of the template. The curing of the repair-surfaces was performed against plexiglass to standardize specimens’ surfaces, eliminate the oxygen-inhibited surface layer and to achieve an initially smooth surface finish. The area to be repaired was finished with abrasive discs (Sof-Lex polishing disc series; coarse, medium, fine, superfine; 3M ESPE). Specimens were cleaned in an ultrasonic bath (Emmi-20HC, eMAG, Salach, Germany) containing deionized water for 10 min to eliminate debris and then were left to air-dry for 24 h at room temperature.

### 4.2. Surface Conditioning Methods

**Figure 8:**
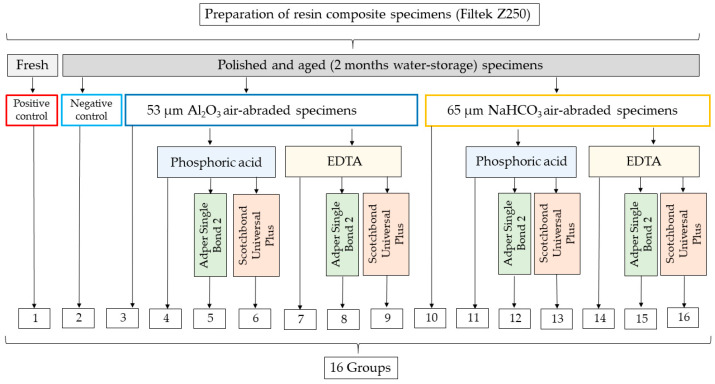
Flowchart of different surface treatment methods of the resin composite samples.

The RBC blocks—except the positive control—were subjected to aging procedure by storage in 45 °C deionized water (Cultura incubator, Ivoclar Vivadent, Schaan, Liechtenstein) for 8 weeks prior to surface conditioning, simulating the oral conditions, where the RBC suffers structural/chemical changes. The specimens received the following surface conditioning treatments (Figure 8):

Group 1. Specimens without polishing, aging and surface conditioning served as positive control.

Group 2. Polished and aged specimens without surface conditioning served as negative control.

Group 3. Polished and aged specimens were air-abraded (Aquacare Twin, Velopex International, London, UK) with 53 μm aluminum oxide particles (Al_2_O_3_, AquaAbrasion, Velopex International, London, UK). The tip of the handpiece was positioned vertically to the surface at a distance of 10 mm over the course of 10 s with a pressure of 4 bar and 90° inclination. The remnant particles were removed using an air-water syringe and then the specimen surface was dried with oil-free air. 

Group 4. The conditioned surface of samples treated as described for Group 3 was cleaned using 35% phosphoric acid (H_3_PO_4_) for 15 s and the etchant was thoroughly rinsed with an air-water syringe for 30 s. The specimen surface was dried with oil-free air.

Group 5. After the procedure as described in Group 4, a thin layer of two-step etch-and-rinse adhesive (Adper Single Bond 2) was applied with circular brushing motion with a disposable applicator for 20 s. The surface was dried with oil-free air-water syringe for 10 s and light-cured for 10 s.

Group 6. After the procedure as described in Group 4, a thin layer of universal adhesive (Scotchbond Universal Plus Adhesive) was applied with circular brushing motion onto the air-abraded surface with a disposable applicator for 20 s and left for 20 s. The surface was then dried with oil-free air-water syringe for 10 s and light-cured for 10 s. 

Group 7. The conditioned surface of samples treated as described for Group 3 was cleaned using 15% ethylene diamine tetra-acetic acid solution (EDTA). The solution was applied via drain tube onto the specimen surface and left for 1 min, then was thoroughly rinsed with an air-water syringe for 30 s. The specimen surface was dried with oil-free air. 

Group 8. A thin layer of Adper Single Bond 2 was applied with the above detailed method to the conditioned surface of samples treated as described for Group 7. 

Group 9. A thin layer of Scotchbond Universal Plus Adhesive was applied with the above detailed method to the conditioned surface of samples treated as described for Group 7.

Group 10. Polished and aged specimens were air-polished (Aquacare Twin, Velopex International, London, UK) with 65 μm sodium bicarbonate particles (NaHCO_3_ AquaPolishing, Velopex International, London, UK). The tip of the handpiece was positioned vertically to the surface from a distance of 4 mm over the course of 10 s with a pressure of 4 bar and 45 degrees of inclination, according to the manufacturer’s recommendation. The remnant particles were removed using an air-water syringe, and then the specimen surface was dried with oil-free air.

Group 11. The conditioned surface of samples treated as described for Group 10 was cleaned using 35% phosphoric acid with the above detailed method.

Group 12. A thin layer of Adper Single Bond 2 was applied with the above detailed method to the conditioned surface of samples treated as described for Group 11. 

Group 13. A thin layer of Scotchbond Universal Plus Adhesive was applied with the above detailed method to the conditioned surface of samples treated as described for Group 11.

Group 14. The conditioned surface of samples treated as described for Group 12 was cleaned using 17% EDTA with the above detailed method.

Group 15. A thin layer of Adper Single Bond 2 was applied with the above detailed method to the conditioned surface of samples treated as described for Group 14. 

Group 16. A thin layer of Scotchbond Universal Plus Adhesive was applied with the above detailed method to the conditioned surface of samples treated as described for Group 14.

### 4.3. Repair Restoration

All substrate RBC specimens were individually placed into the plexiglass mold and repaired with the same type of RBC. The repair RBC was condensed well in 1.5 mm thick increments onto the substrate surfaces and light-cured for 40 s per increment. The incremental packing was continued until the remaining 8 mm × 8 mm wide, 10 mm long recess in the mold was completely filled. In the positive control group (Group 1), the RBC was applied directly onto the fresh substrate. All surface treatment procedures and RBC applications were performed by a single experienced operator in accordance with the manufacturers’ instructions. The specimens were removed from the mold and were stored in 45 °C deionized water for 1 week prior to testing procedures.

### 4.4. Microtensile Bond Strength (μTBS) Testing

**Figure 9:**
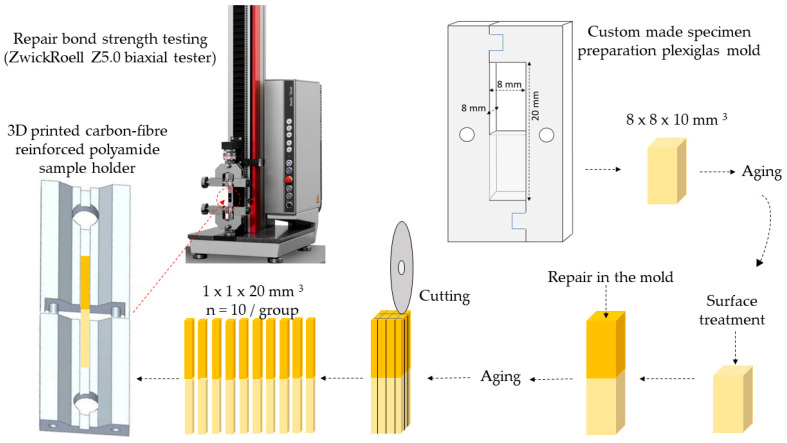
Schematic flowchart of the steps of sample preparation for microtensile bond strength measurements.

The fabricated RBC blocks were secured to a flat polymer holder using a flowable RBC (Filtek Ultimate, 3M ESPE, St. Paul, MN, USA) prior to being mounted on a milling machine (SIEG SX3, Shanghai SIEG Machinery Co., Shanghai, China). The blocks were then longitudinally sectioned in two directions perpendicular to the interface using a water-cooled, diamond-coated cut-off wheel (Superflex fine, Edenta, Lustenau, Austria) at low speed, yielding rectangular sticks from the center of each specimen. To obtain uniform specimens with dimensions of 1 mm × 1 mm × 20 mm, a Newall B60 Digital Readout measurement system (Newall Measurement Systems Ltd., Leicester, UK) was applied. The test specimens were cleaned in an ultrasonic bath with distilled water for 2 min. After cleaning, the specimens were examined under an operating microscope (Leica M320, Leica, Wetzlar, Germany) at 40× magnification for voids and imperfections. Only flawless sticks were tested. The final dimensions of each stick were verified using a screw thread micrometer with 0.001 mm accuracy (Mitutoyo, Tokyo, Japan) to calculate the bonding area. Ten intact sticks were obtained for each group. All specimens were individually bonded (Super Bond, Henkel Loctite, Düsseldorf, Germany) to a custom-made microtensile bond strength jig. The jigs were 3D printed (Markforged X7, Montreal, QC, Canada) from Markforged Onyx using continuous carbon fiber forcing (Marfkorged X7, Montreal, Quebec, Canada). The jigs (0.04 N weight) consisted of two parts, which were secured to each other with metal pins allowing a tension-free fit. A 2 mm gap was formed between the two jig halves by means of a spacer, and hence the bond strength at the interface between the aged and repair RBC could be examined without tension. The specimen fixed in the jig was subjected to load in a biaxial testing machine (Zwick/Roell Z5.0, ZwickRoell, Ulm, Germany) equipped with a 5 kN load cell, which was calibrated with a PCE-BS 3000 laboratory scale (PCE Holding GmbH, Hamburg, Germany) before testing. The strength tests were carried out at a 2 N pre-load and 0.5 mm/min crosshead speed. The applied force (N) was recorded. The applied load at failure (N) divided by the adhesive interface (mm^2^) gave the microtensile bond strength in MPa. The operator of the testing machine was blinded to the tested samples. Figure 9 illustrates the sample preparation process for µTBS tests.

### 4.5. Failure Type Analysis

After µTBS tests, five specimens were randomly selected and subjected to failure mode analysis under a scanning electron microscope (SEM) (JEOL JSM-IT500HR, JEOL, Tokyo, Japan) at 65× magnification, using the backscattered electron mode. Failure modes were recorded as adhesive (between substrate and repair RBC), cohesive (within the substrate or the repair RBC), or mixed (both adhesive and cohesive).

### 4.6. Scanning Electron Microscopic Analysis

Further RBC specimens were prepared (n = 5) in the custom-made template and divided into 7 groups, as follows: surface conditioned with aluminum oxide air-abrasion; surface conditioned with aluminum oxide air-abrasion and cleaned with phosphoric acid; surface conditioned with aluminum oxide air-abrasion and cleaned with EDTA; surface conditioned with sodium bicarbonate air-polishing; surface conditioned with sodium bicarbonate air-polishing and cleaned with phosphoric acid; surface conditioned with sodium bicarbonate air-polishing and cleaned with EDTA; and control sample without surface treatment. During the surface conditioning procedure, half of the sample surface was covered with a metal strip to avoid air-abrasion/air-polishing. This method provided specimens where the treated and untreated surfaces were directly comparable and the surface effect of the abrasives could be judged. The specimens were sputter-coated with gold to a thickness of approximately 5 nm in a vacuum evaporator (Auto-fine coater, JFC-1300, JEOL, Tokyo, Japan) in order to analyze the surface morphology under SEM (JEOL JSM-IT500HR, JEOL, Tokyo, Japan). Micrographs were taken at standardized magnifications (350×, 1000×) in order to document the surface texture created by the different mechanical treatments. Additionally, an energy-dispersive X-ray spectroscope (FEG-SEM-EDS; JEOL JSM-IT500HR, JEOL, Tokyo, Japan) was used to collect detailed elemental information along with electron microscopy images to enable the chemical characterization of the surface-treated specimens. Five scan plots were taken at regular distances of 15 µm along a scanning line of the SEM image. The electron beam with 30 kV landing voltage struck the conducting sample’s surface in high vacuum mode at 700× magnification. The element analysis by the Point-and-ID-method was assessed. 

### 4.7. Statistical Analysis

Sample size calculation was performed according to the results of a previous study [31]. 

The following formula was used (Equation (1)):

(1) $$n=\frac{{\left(z_{1-\frac{\alpha }{2}}+z_{1-\beta }\right)}^{2}{\left(s_{1}+s_{2}\right)}^{2}}{(M_{1}-{M_{2})}^{2}}$$

z = standard score; α = probability of Type I error = 0.05; z_1−α/2_ = 1.96; β = probability of Type II error = 0.20; 1 − β = the power of the test = 0.80; z_1−β_ = 0.84, M_1_ = 25.37, s_1_ = 8.59, M_2_ = 10.45 and s_2_ = 4.66. By adopting an alpha (α) level of 0.05 and a beta (β) level of 0.20 (power = 80%), the predicted sample size (n) was found to be a total of 6.18 samples per group. To perform the µTBS test, a sample size of n = 10 per group was selected.

The statistical analyses were performed with SPSS (Version 26.0; IBM, Armonk, NY, USA). The Kolmogorov–Smirnov test was applied to test the normal distribution of the data, followed by a parametric statistical test. The µTBS of the investigated groups was compared with one-way analysis of variance (ANOVA). Tukey’s post hoc adjustment was used for multiple comparison. General linear model and partial eta-squared statistics were used to test the influence and describe the relative effect size for the surface treatment, cleaning method and applied adhesive system as independent factors. The partial eta squared described the proportion of the variability in the dependent measure (µTBS) that was attributable to the independent factor. *p* values below 0.05 were considered statistically significant.

## 5. Conclusions

Within the limitations of this study, analyzing the different variables influencing the repair process of aged RBC has led to the following conclusions:

- A diverse combination of the applied surface preparation, cleaning method and intermediate adhesive layer significantly contributes to the repair bond strength achieved.
- The widely available and routinely applied prophylactic cleaning method, namely sodium bicarbonate air-polishing followed by EDTA-cleaning and universal adhesive application, can provide a similar RBC repair bond strength to that of the commonly recommended aluminum oxide air-abrasion with or without phosphoric acid cleaning and/or adhesive application.
- For clinical relevance, sodium bicarbonate air-polishing followed by EDTA cleaning and universal adhesive application could be a viable alternative in the RBC repair protocol.

## Figures and Tables

**Table 1:** Relative effect size of surface treatment, adhesive system, cleaning method factors and their interactions on the microtensile bond strength of repaired resin composites. General linear model and partial eta-squared statistics (ƞ^2^).

Factor	*p*-Value	ƞ^2^	Observed Power
Surface treatment (ST)	<0.001	0.516	1.000
Cleaning method (CM)	0.172	0.053	0.364
Adhesive system (AS)	0.034	0.099	0.641
ST × CM	<0.001	0.641	1.000
AS × CM	0.161	0.055	0.377
ST × AS	0.264	0.040	0.283
ST × AS × CM	0.24	0.043	0.301

**Table 2:** Composition and manufacturer details of the materials used in the study.

Product	Composition	Lot Number	Manufacturer
Filtek Z250	Bis-GMA, UDMA, Bis-EMA, TEGDMA, 0.6 µm zirconia-silica, 78 wt%, CQ, tertiary amine	9438725	3M ESPE, St. Paul, MN, USA
Adper Single Bond 2	BisGMA, HEMA, UDMA, glycerol 1,3 dimethacrylate, DPIHP, EDMAB, ethanol, water, copolymer of polyacrylic and polyitaconic acids, 5 nm filler	NE70992	
Scotchbond Universal Plus Adhesive	BisGMA, HEMA, 1,10-decanediyl bismethacrylate, HEMA-phosphate, MDP, CQ, EDMAB, ethanol, water, copolymer of polyacrylic and polyitaconic acids, silane, filler	7730432	
Ultra-Etch	Phosphoric acid (<40 wt%), cobalt aluminate blue spinel (<1 wt%), siloxane (<1 wt%)	311211	Ultradent, South Jordan, UT, USA
Endo-Solution	EDTA-Na2 (15 wt%), sodium hydroxide (1 wt%)	2401221	Cerkamed, Stalowa Wola, Poland

BisGMA: bisphenol A diglycidil ether dimethacrylate; UDMA: urethane dimethacrylate; BisEMA: bisphenol A polyethylene glycol diether dimethacrylate; TEGDMA: triethylene glycol dimethacrylate; HEMA: hydroxyethyl methacrylate; DPIHP: diphenyliodonium hexafluorophosphate; MDP: methacryloyloxydecyl dihydrogen phosphate;CQ: camphoroquinone; EDMAB: ethyl 4-dimethyl aminobenzoate, EDTA-Na2: ethylene diamine tetraacetic acid.

## Data Availability

The data that support the findings of this study are available from the corresponding author upon reasonable request.

## Supplementary Materials

The following supporting information can be downloaded at: https://www.mdpi.com/article/10.3390/ijms241411568/s1.
